# An Intuitive Model of Bystander Responses to Workplace Mistreatment

**DOI:** 10.3390/bs16040477

**Published:** 2026-03-24

**Authors:** Qiuyue Shao, Ke Zhang, Xiaoping Zhao

**Affiliations:** 1 School of Economics and Management, Tongji University, Shanghai 200070, China; 2110090@tongji.edu.cn; 2 School of Accounting, Shanghai Lixin University of Accounting and Finance, Shanghai 201620, China; 3 Department of Enterprise Management, Research Institute of Economics and Management, Southwestern University of Finance and Economics, Chengdu 610074, China; zhaoxp@swufe.edu.cn

**Keywords:** workplace mistreatment, bystander responses, bystander intervention, moral intuition, mistreatment prototypes

## Abstract

Our paper presents an intuitive model of bystander intervention to workplace mistreatment. Drawing on the literature on moral intuition, our paper proposes (1) that bystanders match an observed conduct to mistreatment descriptions (the first type of mistreatment prototypes), and (2) that bystanders make intuitive judgments and take immediate interventions when intervention prescriptions (the second type of mistreatment prototypes) exist in their long-term memory. Our paper also argues that bystanders’ intuitive judgments and interventions depend on the accessibility of their mistreatment prototypes, which are formed through learning mechanisms. Our paper contributes to the literature on bystander responses to workplace mistreatment.

## 1. Introduction

Given the rise of mistreatment (e.g., abuse, bullying, harassment, and violence) in the workplace and its severe consequences for organizations and their members (Vranjes & Lyubykh, 2021), researchers have devoted increasing attention to understand bystander intervention (Vranjes et al., 2025; Zhong et al., 2023). Many studies argue that bystanders’ intervention results from a rational and deliberative process, in which bystanders notice a mistreatment event, evaluate the severity for the victims’ situation, consider whether an action is needed, decide if they have a responsibility, choose what form of actions to take, and understand how to implement the choice safely (Coyne et al., 2019). Others emphasize a cognitive appraisal process, suggesting that mistreatment triggers cognitive appraisals that in turn result in emotional responses (e.g., D’Cruz & Noronha, 2011; Ryan & Wessel, 2012).

Though insightful, prior work provides only a partial explanation of bystander intervention. Empirical studies and everyday experience show that individuals often make judgments and respond to external stimuli automatically and rapidly via intuition (Kahneman, 2003). This suggests that bystanders may also respond to mistreatment intuitively, not just rationally. For example, Skarlicki and Rupp (2010) report that bystanders are more likely to retaliate against perpetrators when they rely on a moral intuitive process rather than on a rational reasoning process.

Drawing from the moral intuition literature (Haidt, 2001; Weaver et al., 2014), our paper proposes an intuitive model in which bystanders’ judgments and intervention are generated intuitively. Central to our model are cognitive prototypes—the previously formulated patterns regarding workplace mistreatment are stored in memory. We focus on two types of mistreatment prototypes—mistreatment descriptions and intervention prescriptions (Reynolds, 2006). Through an associative process, bystanders can establish a connection between an observed conduct and mistreatment descriptions when the perceived fit is sufficiently strong. This connection leads to the automatic recognition of the conduct as mistreatment. Bystanders’ judgment of a conduct as mistreatment triggers their sympathy, which in turn motivates bystanders with intervention prescriptions to intervene immediately. Our model also outlines the conditions that can prevent this intuitive process from initiating or completing. Finally, our paper argues that the two mistreatment prototypes develop through two learning mechanisms, including personal experiences and social influence.

The paper proceeds as follows. Section 2 reviews the literature on bystander responses to workplace mistreatment. Section 3 presents our intuitive model. In Section 4, we discuss the learning mechanisms of the development of mistreatment prototypes. Finally, Section 5 concludes with our model’s theoretical contributions and practical implications, limitations, and suggestions for future research.

## 2. Literature Review of Bystander Responses

### Bystander Responses to Workplace Mistreatment

In the pioneering work, Latané and Darley (1970) propose a rational process in which bystanders (1) notice mistreatment, (2) evaluate it by assessing the severity of harm, moral intensity, social influence, and actor-target relationship, (3) attribute the blame, and (4) take a response. Later, scholars propose conceptual models and conduct empirical analyses based on this rational reasoning perspective to delineate bystander responses to workplace mistreatment.

Conceptually, Ashburn-Nardo et al. (2008) propose that bystanders interpret an event as discriminatory, feel responsible, identify a solution, and finally implement that solution. Skarlicki and Kulik (2004) argue that abuse invokes strong emotions, which drive bystanders to intervene. However, some models argue that bystanders do not always respond pro-socially. According to Bowes-Sperry and O’Leary-Kelly (2005), bystanders’ prosocial responses toward the target are contingent on the ambiguity of conduct, moral intensity of the incident, social influence, actor-victim relationships, social appropriateness of intervention, and social identity. In addition, Elkins and Velez-Castrillon (2008) argue that bystanders’ pro-social responses depend on several factors, such as victims’ responses, severity of harm, pervasiveness, and responsibility attributions.

Besides conceptual models, empirical studies have also been conducted to examine bystander responses. Priesemuth (2013) employs a multi-method approach, conducting one laboratory experiment and two field studies where employees report on observed abusive supervision and supervisors rate subsequent employee prosocial behaviors. Priesemuth (2013) reports that employees who observe the abuse are likely to exhibit prosocial actions towards the victim, but this relationship is highly context-dependent. Ryan and Wessel (2012) conduct two studies: a retrospective study where participants described real witnessed incidents of sexual harassment and a scenario-based experiment where participants read a vignette involving a coworker making a derogatory joke. The two studies find that observers are more likely to intervene against sexual harassment when the harassment is direct, the target’s orientation is known, they have a closer relationship to the target, and they perceive the costs of intervention to be low.

Conducting five scenario-based experiments with undergraduate students, Diekmann et al. (2013) use a written vignette where a job candidate is asked sexually harassing questions during an interview and responds passively by answering them. After reading the scenario, participants first forecast their own hypothetical responses and then evaluate the victim; the experiments employ between-subject designs. The experiments suggest that observers’ non-intervention is primarily driven by their own flawed behavioral forecasts—where they overestimate how confrontational they would be—coupled with a failure to consider the victim’s situational motivations (e.g., fear of retaliation or job loss) and a tendency to attribute passivity to the victim’s character rather than external constraints. In addition, Chui and Dietz (2014) conduct two experimental studies in Switzerland to examine when bystanders perceive harm and feel compelled to intervene after witnessing workplace incivility toward a woman. Bystanders consistently perceive more harm when the female target reacts by crying rather than laughing or neutrally, but this does not always translate into a stronger perceived need to intervene.

In summary, previous articles primarily describe bystander responses as the product of rational reasoning processes (Skarlicki & Kulik, 2004; Skarlicki & Rupp, 2010). Moreover, these articles identify several factors that can influence the likelihood of bystanders responding pro-socially. While informative, prior articles have a significant gap. Specifically, it overlooks the role of intuition in influencing bystanders’ judgments and intervention. In fact, individuals frequently make judgments and behave intuitively (Adolphs, 2009). To address this gap, this paper draws on the moral intuition literature (Reynolds, 2006; Sonenshein, 2007) to propose a model in which intuition generates emotionally laden judgments and prompts immediate intervention.

## 3. An Intuitive Model of Bystander Responses to Workplace Mistreatment

### 3.1. Moral Intuition as an Associative Process Based on Prototypes

Haidt and Bjorklund (2008, p. 188) define moral intuition as “the sudden appearance in consciousness, or at the fringe of consciousness, of an evaluative feeling (like–dislike, good–bad) about a person or event without any conscious awareness of having gone through steps of weighing evidence, crafting evaluative arguments, or inferring a conclusion.” Moral intuition entails both intuitive judgments (Dane & Pratt, 2007).

To generate intuitive judgments, moral intuition relies on an associative process (Weaver et al., 2014), where environmental stimuli are matched against deeply held nonconscious cognitive schemas, or prototypes (Reynolds, 2006). The automatically activated associations between the stimuli and the prototypes result in intuitive and emotionally charged judgments (Hodgkinson & Sadler-Smith, 2018). Such judgements will further trigger immediate actions (Malle, 2021; Sonenshein, 2007).

### 3.2. The Intuitive Process of Bystander Responses to Workplace Mistreatment

#### 3.2.1. Mistreatment Prototypes

For intuitive judgments and immediate intervention to occur, bystanders must have two mistreatment prototypes in memory (Reynolds, 2006). These prototypes are exemplars of concepts, issues, and situations related to workplace mistreatment.

**Figure 1:**
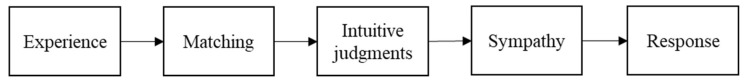
The prototype-based intuitive model of bystander responses to workplace mistreatment.

The first prototype is mistreatment descriptions—the cognitive schemas depicting the attributes of workplace mistreatment. The second prototype is intervention prescriptions—the schemas for appropriate responses (Reynolds, 2006). Take bystanders’ intuitive responses to sexual harassment as an example. Here, mistreatment descriptions define what constitutes harassment, while mistreatment prescriptions specify appropriate intervention methods. The descriptions enable bystanders to rapidly identify harassment and feel immediate sympathy. Sympathetic bystanders with the corresponding prescriptions can intervene immediately; those without them may need to deliberate (Bowes-Sperry & O’Leary-Kelly, 2005; Fischer et al., 2011; Mujal et al., 2021). We use Figure 1 to demonstrate our model.

In this model, *Experience* refers to an observation (i.e., bystander mistreatment observation), *Matching* refers to the evaluative process fitting the experience with the first prototype (i.e., mistreatment descriptions), *Intuitive Judgments* refer to the recognition of an experience as bystander mistreatment, *Sympathy* refers to the emotional concern for other persons, and *Response* refers to an intervention response to the experience.

#### 3.2.2. Bystanders’ Intuitive Judgments

This section details how bystanders generate intuitive judgments of mistreatment via an associative process. The process begins when the bystanders observe a conduct (*Experience*). Then, *Matching* is triggered, in which bystanders match observed conduct against mistreatment descriptions stored in their memory. This process occurs automatically, where bystanders estimate the degree of fit between the conduct’s attributes and mistreatment descriptions. The fit will activate an association between the observed conduct and mistreatment (Cerulo et al., 2021; Skarlicki & Rupp, 2010). When the degree of fit is higher, a stronger association will be activated, and bystanders are more likely to intuitively perceive the observed conduct as mistreatment; conversely, a lower degree of fit activates a weaker association, and bystanders are less likely to perceive the observed conduct as mistreatment (Reynolds, 2006).

**Proposition** **1.** *The fit between the conduct’s attributes and mistreatment descriptions is positively related to bystanders’ mistreatment judgments.*

Above, we emphasize the importance of the fit between the conduct’s attributes and mistreatment descriptions to activate bystanders’ intuitive judgments. We further argue that accessibility of mistreatment descriptions serves as one important condition for that activation. To activate intuitive judgments, the prototypes must be accessible (Killen & Dahl, 2021; Wheeler et al., 2005). Therefore, we propose that when the mistreatment descriptions are more accessible, bystanders are more likely to rely on the mistreatment descriptions to make intuitive judgments.

**Proposition** **2.** *The positive relationship between fit and mistreatment judgment is moderated by the accessibility of mistreatment descriptions, such that the relationship is stronger when mistreatment descriptions are more accessible.*

#### 3.2.3. Bystanders’ Sympathy

Having established that bystanders can make intuitive judgments about mistreatment, a critical question arises: What are the consequences of these judgments? The moral intuition literature consistently notes that intuitive judgments are accompanied by moral emotions, defined as those linked to the interests or welfare of others or society (Dhanani & LaPalme, 2019; Haidt, 2001). Moral emotions, in turn, trigger their immediate actions (Greenbaum et al., 2020; Tangney et al., 2007). In our paper, we argue that sympathy is triggered when conduct is intuitively judged as mistreatment.

As a moral emotion, sympathy refers to an emotional concern for other persons (Loewenstein & Small, 2007). Often, sympathy arises even without a personal stake in the event (Herry et al., 2021). When intuitive judgments of mistreatment are formed, bystanders can immediately feel sympathy. Although not directly harmed, bystanders can understand and feel the victim’s suffering. Moreover, bystanders view workplace mistreatment as violating widely accepted moral rules regarding how people should be treated (Gloor et al., 2024; Jennings et al., 2024), and thus they develop sympathy for the victim (Haidt, 2003). Therefore, we propose that bystanders will experience sympathy when they intuitively judge a conduct as mistreatment.

**Proposition** **3.** *Bystanders’ intuitive judgment of mistreatment is positively related to their sympathy for the victim.*

While Proposition 3 posits a direct link between intuitive judgments and sympathy, this emotion is not generated in a vacuum. Bystanders interpret events through the lens of their social relationships. Consequently, the strength of sympathy is likely contingent upon the bystander’s perceived connection to the victim.

Sympathy is influenced by ingroup-outgroup identification (Carlson et al., 2024)—the awareness of belonging to a group (Shafaei et al., 2024). Stronger identification with a group fosters a greater sense of responsibility to support ingroup members (Coyne et al., 2019) and leads to greater concern for them than for outgroup members (Steffens et al., 2017). Consequently, sympathy increases with the bystander’s felt connections to the victim (Erlandsson et al., 2015). That is, bystanders who view the victim as an ingroup member will experience greater sympathy than bystanders who do not regard the victim as a member of their group.

**Proposition** **4.** *The positive relationship between intuitive judgments and sympathy will be stronger for a victim perceived as an ingroup member than an outgroup member.*

In addition to ingroup-outgroup identification, bystanders’ confidence in their intuitive judgments is likely to moderate the relationship between bystanders’ mistreatment judgments and sympathy. People generally strive to make accurate judgments (Brashier & Marsh, 2020; Lyons et al., 2021). When bystanders have low confidence in their intuitive judgments, the intuitive process is interrupted, and they are less likely to feel sympathy (Hurteau et al., 2020).

The low confidence can have two sources. First, bystanders have engaged in the intuitive process but find that the fit between the attributes of the observed conduct and the mistreatment descriptions is low. As a result, bystanders are uncertain whether the observed conduct is mistreatment. Second, bystanders have intuitively recognized a conduct as mistreatment but are uncertain whether it is appropriate in their organizations. In both cases, low confidence mitigates the relationship between intuitive judgment of mistreatment and sympathy.

**Proposition** **5.** *The positive relationship between intuitive judgments and sympathy will be stronger when bystanders have higher confidence in their judgments.*

#### 3.2.4. Bystanders’ Responses

Bystander intervention to mistreatment can be immediate, allowing bystanders to remove the victim from the situation, interrupt the incident, instruct the perpetrator to stop the mistreatment, and/or encourage other bystanders to intervene in the conduct. As bystanders’ intuitive judgments can lead to sympathy, we further propose that sympathy can serve as an automatic motivational driver for immediate intervention (Warner et al., 2024).

Sympathy can motivate individuals to help others or alleviate the sufferings of others (Jeong et al., 2023). Moreover, the motivational effect of sympathy depends on its level of arousal (Baas et al., 2008). More specifically, low levels of arousal lead to inactivity and avoidance, moderate levels of arousal result in rational reasoning and deliberative behaviors, and high levels of arousal motivate individuals to take immediate actions. We thus suggest that sympathy in high levels of arousal is more likely to trigger bystanders’ immediate intervention. In contrast, sympathy in low or moderate levels of arousal is less likely to motivate immediate intervention.

**Proposition** **6.** *The arousal level of bystanders’ sympathy is positively related to bystanders’ immediate intervention.*

Proposition 6 establishes sympathy as a motivator for immediate intervention. However, sympathy alone may be insufficient for action. To act immediately, bystanders must also know how to intervene. As noted above, the second mistreatment prototype—intervention prescriptions—represents cognitive schemas for appropriate intervention (Reynolds, 2006). With these prescriptions stored in memory, bystanders know how to act and hence can intervene immediately (Mujal et al., 2021). In contrast, bystanders without these prescriptions stored in memory are less likely to display immediate intervention behaviors.

**Proposition** **7.** *The positive relationship between sympathy and immediate intervention is stronger when mistreatment prescriptions are more accessible.*

In addition to intervention prescriptions, organizational ethical climate can moderate the relationship between bystanders’ sympathy and their immediate intervention. Organizational ethical climate reflects members’ collective beliefs concerning what behaviors are appropriate or inappropriate in an organization (Kuenzi et al., 2020). In an organization with a stronger organizational ethical climate, workplace mistreatment is inappropriate and more discouraged. In addition, a strong ethical climate promotes prosocial behaviors such as interventions (Vardaman et al., 2014; Wang & Yen, 2023). Consequently, sympathetic bystanders in a strong ethical climate are more likely to intervene immediately.

**Proposition** **8.** *The positive relationship between sympathy and immediate intervention is stronger when the ethical climate is stronger.*

## 4. The Development of Mistreatment Prototypes

So far, we have discussed the intuitive model and emphasized the role of the two mistreatment prototypes (Propositions 1–8 in Figure 1). However, an important question remains: how do these mistreatment prototypes develop? We answer this question in this section. We propose that the two types of mistreatment prototypes—mistreatment descriptions and intervention prescriptions—can develop via two learning mechanisms: individual experience and social influence.

### 4.1. Individual Experience

Intuitive prototypes can be developed, reinforced, and modified through a feedback loop of individual experience (Dane & Pratt, 2007). The loop begins with an intuitive judgment, accompanied by bystanders’ immediate responses. Furthermore, bystanders engage in post hoc reasoning—an effortful search for arguments to support or challenge the intuitive judgments and responses (Haidt, 2001; Killen & Dahl, 2021). In the post hoc reasoning, bystanders may analyze the feedback from the victims or others (Alerasoul et al., 2022; Lei et al., 2016). Positive feedback reinforces existing prototypes, while negative feedback can modify them.

For example, recognizing bullying and intervening immediately can provide positive feedback to bystanders, leading them to experience positive emotions such as pride (Banyard et al., 2021). Consequently, the positive feedback and the experienced positive emotions can reinforce bystanders’ established prototypes of bullying. Bystanders would rely more on their mistreatment prototypes when they observe bullying in the future. In contrast, bystanders who fail to recognize an abuse as mistreatment may later observe the victim’s distress as negative feedback, potentially feeling regret (Gilovich & Medvec, 1995). This feeling is likely to trigger post hoc reasoning, during which bystanders analyze the negative feedback. Such reflection can challenge their initial judgments. Consequently, bystanders may revise their mistreatment descriptions, leading them to be more likely to perceive similar conduct as mistreatment in the future.

In summary, bystanders gain more experience and become “experts” of workplace mistreatment when they learn more from the positive and negative feedback (Kuntz & Searle, 2023). Thus, mistreatment prototypes become dynamic as they can be developed, reinforced, and modified. The dynamic prototypes can further alter bystanders’ judgments and responses.

**Proposition** **9.** *Positive feedback following an intuitive response reinforces existing mistreatment prototypes, whereas negative feedback leads to their modification.*

### 4.2. Social Influence

Intuitive prototypes are subject to social influence within an organization (Weaver et al., 2014). In an organization, organizational cultures and norms shape individuals’ perceptions regarding right and wrong (Dedeke, 2015) and hence form individuals’ mistreatment prototypes (Hodgkinson & Sadler-Smith, 2018; McManus, 2021). Among many organizational cultures and norms, organizational ethical climate is particularly relevant to the development of mistreatment prototypes.

As noted above, organizational ethical climate reflects organizational members’ collective beliefs concerning what behaviors are appropriate or inappropriate in an organization (Kuenzi et al., 2020). Bystanders can directly learn from it what constitutes mistreatment and appropriate responses (Treviño et al., 2006). Moreover, bystanders can indirectly learn about whether a conduct is mistreatment and how to appropriately respond through observing other organizational members’ moral behaviors and reactions to mistreatment that are mainly shaped by organizational ethical climate (Collins et al., 2021; Kuntz & Searle, 2023).

**Proposition** **10.** *Bystanders are more likely to develop mistreatment prototypes in organizations with a stronger organizational ethical climate.*

## 5. Discussions and Conclusions

### 5.1. Theoretical Implications

#### 5.1.1. Contributions to Studies on Bystander Responses and Intervention

Scholars have devoted considerable attention to investigating bystander responses to workplace mistreatment. Previous studies largely focus on the deliberative processes of bystander responses. To complement the existing literature, we propose a moral-intuition model that emphasizes two mistreatment prototypes and shows how they develop through learning mechanisms.

Workplace mistreatment frequently unfolds under conditions characterized by ambiguity, social risk, and limited time for reflection. In such contexts, bystanders often cannot engage in extended moral reasoning. Instead, intervention may depend on whether observed conduct is immediately recognized as mistreatment through intuitive matching processes. Our intuitive process model therefore shifts theoretical attention from decision making after recognition to recognition itself. From this perspective, intervention failure may not necessarily reflect moral disengagement or unwillingness to help, but rather the absence or inaccessibility of cognitive prototypes that enable rapid identification of mistreatment. This reconceptualization advances the bystander literature by introducing intuition as a foundational mechanism linking judgment, emotion, and action. Bystander responses can thus be understood as emerging from automatic associative processes that generate moral judgments and emotional reactions prior to conscious evaluation. By highlighting intuitive recognition as an antecedent to intervention, our model complements deliberative perspectives and provides a more psychologically realistic account of how bystanders respond in real organizational settings.

Furthermore, across multiple domains—including managerial decision making, medical expertise, emergency response, and ethical judgment—intuition has been shown to enable effective action under uncertainty and time pressure. Experts in these domains rely on stored cognitive schemas developed through experience, allowing them to recognize meaningful patterns rapidly and respond without extensive deliberation. Bystanders confronting workplace mistreatment face similar cognitive conditions. They must interpret socially complex situations, assess moral meaning, and act despite incomplete information and potential interpersonal consequences. By applying intuition-based theories to bystander behavior, our model positions intervention as a form of socially situated expertise grounded in learned prototypes. This perspective helps explain why experienced employees, trained observers, or individuals embedded in strong ethical environments often intervene more quickly and confidently than others.

Finally, our model advances this literature by specifying the cognitive mechanisms through which intuitive bystander responses occur. Prior research has identified contextual and relational factors influencing intervention likelihood, yet less attention has been devoted to how individuals come to perceive an event as mistreatment in the first place. By distinguishing between mistreatment descriptions and intervention prescriptions, we introduce a prototype-based explanation linking perception and action. This distinction clarifies several persistent puzzles in bystander research. First, individuals may recognize harm yet fail to act because intervention prescriptions are absent or inaccessible. Second, organizational environments may influence intervention not only by shaping behavioral norms but also by shaping cognitive recognition processes. Ethical climates therefore affect whether situations are intuitively perceived as morally actionable events rather than merely influencing post-recognition decisions.

#### 5.1.2. Contributions to Studies on Moral Intuition

Our paper also contributes to the moral intuition literature. First, to address how intuitive judgments and immediate behaviors are generated, our model highlights the roles of two types of mistreatment prototypes and suggests that mistreatment descriptions matter to intuitive judgments and intervention prescriptions matter to immediate behaviors.

Second, we explain how individual experiences and organizational ethical climate shape mistreatment prototypes, thus adding new insights into the understanding of the development of moral intuition (Dedeke, 2015; Wang & Yen, 2023). Moreover, we also emphasize that moral intuition could be shaped by organizational-level factors such as organizational ethical climate, complementing existing understanding that moral intuition is influenced by societal-level factors (Haidt, 2001, 2012).

#### 5.1.3. Contributions to Research on Bystander Judgment and Decision Making

Recognizing the role of intuition carries important implications for how bystander processes should be studied. Much existing research relies on retrospective surveys or vignette-based evaluations that capture participants’ reflective explanations of behavior. However, intuitive judgments often occur rapidly and may be reconstructed through post hoc reasoning. As a result, traditional methods may underestimate the influence of intuitive processes.

Our model suggests the need for methodological approaches capable of capturing real-time cognition and emotional activation. Experimental designs incorporating time pressure, affect priming, behavioral simulations, virtual reality scenarios, and experience sampling methods are particularly well suited to examining intuitive recognition and intervention. Reaction-time measures and longitudinal designs may further illuminate how mistreatment prototypes develop through learning and organizational socialization.

More broadly, our model encourages scholars to examine bystander intervention as a dynamic process unfolding across perception, emotion, and action rather than as a single behavioral outcome. Such approaches may substantially advance understanding of when and why intervention occurs in naturalistic organizational environments.

### 5.2. Practical Implications

Our findings also carry implications for organizational practice and policy aimed at reducing workplace mistreatment. Organizations have realized for a long time that workplace mistreatment has detrimental consequences not only to the victims but also to the organizations (Dhanani et al., 2021; McCord et al., 2018). Traditional intervention initiatives often emphasize encouraging employees to carefully evaluate situations and decide whether to act. In contrast, our model suggests that effective intervention depends on developing shared cognitive prototypes that enable rapid recognition and response. Organizations can therefore strengthen bystander intervention by shaping both mistreatment descriptions and intervention prescriptions. Training programs should provide concrete examples of mistreatment, helping employees recognize subtle or ambiguous behaviors as violations of acceptable conduct. Equally important, organizations should institutionalize clear intervention scripts that reduce uncertainty about how to respond safely and appropriately.

Furthermore, cultivating a strong ethical climate may influence intervention at an earlier cognitive stage than previously assumed. Ethical environments do not merely motivate prosocial action; they help employees internalize prototypes that align intuitive judgments with organizational values. Policies promoting transparency, ethical leadership, and visible responses to misconduct can reinforce learning processes that make intervention increasingly automatic over time. By focusing on recognition and preparedness rather than solely on deliberate choice, organizations may enhance the likelihood that bystanders intervene when mistreatment occurs.

### 5.3. Limitations and Future Research Directions

Our paper has several limitations. First, it focuses exclusively on the intuitive process. Only when with limited cognitive capacity, bystanders are likely to rely on the intuitive process (Cimpian & Salomon, 2014; Trope & Gaunt, 1999). Furthermore, bystanders low in need for cognition are more likely to rely on the intuitive process (Hurteau et al., 2020). Given that our model focuses on the intuitive process, future studies may explore under what conditions the intuitive process is more likely to be triggered. Furthermore, future studies may consider dual models that incorporate both the intuitive and conscious processes to provide a more comprehensive understanding of bystander responses to mistreatment (O’reilly & Aquino, 2011).

Second, our paper does not empirically examine the propositions. Future studies can examine the intuitive process using scenario-based methods (van Gelder et al., 2024), affect priming (Welsh & Ordóñez, 2014), time-pressured decision tasks (Endres et al., 2020), or independent coders rating the degree of intuitions implicit in an interview with participants (McAdams et al., 2008). Experience sampling methodology (ESM) is particularly well-suited, allowing researchers to capture bystanders’ real-time intuitive judgments, emotional states (sympathy), and intervention attempts in naturalistic settings, testing the core links in our process model. Longitudinal studies are also suggested to examine how prototypes develop and change over time.

Finally, our model generally focuses on whether bystander responses could result from intuitive processes. Our model does not speak much about the specific interventions generated by the intuitive process. To extend our model, further studies can examine which specific interventions are involved in the intuitive process.

### 5.4. Conclusions

Our paper presents an intuitive model of bystander responses to workplace mistreatment. We emphasize the role of two mistreatment prototypes (i.e., mistreatment descriptions and intervention prescriptions) and suggest that bystanders’ intuitive judgments and sympathy are generated by an associative process based on mistreatment prescriptions and that sympathy motivates bystanders with intervention prescriptions stored in their memory to display immediate intervention. We further discuss the development of the two mistreatment prototypes and propose that they can be shaped by individual experiences and social influence.

## Data Availability

No new data were created or analyzed in this study. Data sharing is not applicable to this article.
